# Pressure Injury Recurrence After Flap Surgery in Home‐Dwelling Patients With Spinal Cord Injury in Norway: A Retrospective Study

**DOI:** 10.1111/iwj.70211

**Published:** 2025-04-15

**Authors:** Anne Riisøen Selsjord, Lena Leren, Ingebjørg Irgens

**Affiliations:** ^1^ Oslo Metropolitan University Oslo Norway; ^2^ Sunnaas Rehabilitation Hospital Bjørnemyr Norway; ^3^ University of South‐Eastern Norway Drammen Norway

**Keywords:** flap surgery, pressure injury, pressure injury prevention, pressure injury recurrence, spinal cord injury

## Abstract

A retrospective single‐centre study. To investigate the period prevalence of pressure injury recurrence (PIR) and characteristics associated with PIR in the population of persons with spinal cord injury (SCI) who were treated with flap surgery between 2008 and 2019. A spinal cord unit (SCU) in Norway. The study is based on analysis of patient data from the electronic medical record. Crosstabs and logistic regression were used to investigate the potential correlations between the odds of PIR and potential risk characteristics. We identified 54 patients who were treated with flap surgery in the period of interest, and 47 (87%) were men. The mean age for flap surgery was 51 years (SD = 12.7). The occurrence of PIR post‐flap surgery was 46% (*n* = 25). Factors associated with increased risk of PIR were use of manual wheelchair (65% vs. 32%, odds ratio [OR] = 3.9, 95% confidence interval [CI] = 1.06–14.33, *p* = 0.04) when compared with powered wheelchair, and history of PI and flap surgery at the ischial tuberosity (sit bones) (68% vs. 24%, OR = 3.67, 95% CI = 1.01–13.40, *p* = 0.04) compared with all other body locations. Factors associated with decreased risk of PIR were independence in position changes (29% vs. 58%, OR = 0.29, 95% CI = 0.91–0.95, *p* = 0.04) compared with not needing assistance with position changes, tetraplegia (C5–C8) (21% vs. 60%, OR = 0.18, CI = 0.04–0.83, *p* = 0.02) compared with paraplegia, and postoperative follow‐up by the SCU (15% vs. 55%, OR = 0.28, 95% CI 0.03–0.76, *p* = 0.04) compared with no follow‐up from the SCU, as well assistance from personal assistant follow‐up (PAF) (26% vs. 56%, OR = 0.15, 95% CI = 0.03–0.76, *p* = 0.01) compared with not receiving assistance from PAF. The period prevalence of PIR post‐flap surgery in the Norwegian population of people with SCI is high and increased odds of PIR were related to flap surgery on the sit bones. Reduced odds of PIR were related to tetraplegia, powered wheelchair use, follow up by PAF and the SCU. The study is registered in the Open science framework.

**Summary**

- Pressure injury recurrence (PIR) after flap surgery is high in the Norwegian population of individuals with spinal cord injury (SCI).
- Use of manual wheelchair, previous pressure injury, lack of user‐focused personal assistance, and lack of follow‐up from the spinal cord unit (SCU) increased the odds of PIR.
- Postoperative follow‐up from the SCU, in collaboration with the local healthcare services in the community, has the potential for improved continuity of care for home‐dwelling individuals with SCI. However, this requires accurate PI documentation, as well as a person‐centred approach.

## 1 Introduction

People with spinal cord injury (SCI) are at high risk of developing pressure injury (PI) [1, 2, 3], due to impaired motor function, reduced skin sensation, altered circulation and moister [1, 2, 3, 4, 5, 6, 7]. Several studies suggest that individuals with complete SCI are at higher risk of developing PI compared with persons with incomplete SCI [1, 3, 7]. However, findings regarding the relationship between PI risk and the level of SCI are inconsistent. Some studies reported that higher‐level injuries are associated with an increased PI risk [1, 3] while others find lower‐level injuries to be a risk factor for PI [1, 8]. However, no single factor can fully explain the risk of developing PIs. Instead, the risk arises from a complex interplay of factors that increase the likelihood of PI [9]. PI most commonly occurs at bony prominence such as the sacrum, heels, trochanter and buttocks, especially when it comes to sitting‐acquired PI, but the occurrence of PI may also be related to medical devices [2, 3].

The consequences of having a PI are severe, leading to physical and psychological discomfort, social limitations and reduced participation in meaningful activities [1, 3, 4, 7, 8], PIs also frequently result in hospital readmissions, prolonged inpatient stays and increased healthcare costs [6, 10].

Despite various attempts to achieve rapid PI healing, the most severe cases still require surgical intervention [11, 12, 13, 14, 15]. There are various flap‐surgery techniques, but limited information exists on the comparison of different techniques and their success rates in the population of individuals with SCI [16]. Flap surgery is still the treatment of choice for PIs. However, there is a significant risk of pressure injury recurrence (PIR) in the treated body area, post‐surgery [12, 13, 14, 15, 17]. The occurrence of PIR post‐surgery varies across studies [12, 13, 14, 15, 18]. Keys et al. reported a PIR occurrence of 39%, while Morel et al. found it to be 52% for the population of individuals with SCI [14, 15]. A literature search was conducted in May 2024. One systematic review and a few retrospective studies were found. Associated risk factors were both related to biomedical and social aspects [11, 12, 14, 15, 18]. None of the studies examined assistive aids or post‐operative follow‐up. Therefore, more knowledge about the occurrence and risk factors of PIR after flap surgery in people with SCI is warranted.

The three Norwegian spinal cord units (SCUs) have life‐long follow‐up responsibilities for people with SCI. The two SCUs located in the central and western parts of Norway cover approximately 40% of the total population. The SCU in the south‐eastern part covers the remaining 60%. The focus on the prevention of PI by the three Norwegian SCUs increased after the establishment of the Norwegian Patient Safety Programme in 2012. Based on this programme, together with guidelines for the prevention and treatment of pressure ulcers in the population of people with SCI [1, 3, 19], a knowledge‐based programme for a postoperative mobilisation after flap surgery was incorporated in the Norwegian SCUs. In addition, an outpatient telemedicine treatment programme for home‐dwelling individuals with SCI and PI was established in 2012 [20]. However, this programme has so far not included the prevention of PIR post‐flap surgery for home‐dwelling individuals with SCI. Thus, the aim of this study was to establish knowledge about the incidence of PIR after flap surgery in individuals with SCI in Norway, as well as to examine characteristics associated with PIR in the population. The research results may serve as a basis for improving the quality of follow‐up services for home‐dwelling persons with SCI in Norway.

### 1.1 Research Ethics

The Norwegian Data and Telecommunications Authority's safe information flow requirements were followed [21]. There was no need for ethical approval by the National Regional Ethical Committee, as this study was an internal quality improvement project. The study is performed in accordance with the Helsinki Declaration [22].

## 2 Materials and Methods

### 2.1 Setting and Population

All individuals with an acquired SCI, who were above the age of 18 on the date of PI‐related flap surgery, were included. Furthermore, inclusion criteria were PI‐related flap surgery between 1 January 2008 and 1 January 2019 and hospitalised at the SCU post‐flap surgery.

In this region, the number of individuals with SCI treated with flap surgery, due to PI, has varied between 5 and 10 individuals annually. Thus, we chose to examine the population in a 10‐year period to get an acceptable number of individuals included in the study. Exclusion criteria were congenital spinal cord injuries, such as spina bifida/meningomyelocele. Furthermore, we excluded neurological conditions with the potential for spinal cord affection, such as multiple sclerosis and amyotrophic lateral sclerosis.

### 2.2 Study Design

We conducted a single‐centre, retrospective study, with the aim of estimating the period prevalence of PIR and investigating the association between potential risk factors and PIR in people with SCI post‐flap surgery. Information available from the electronic medical records (EMR) at the SCU was evaluated to identify individuals and retrieve data regarding potential risk factors.

### 2.3 Data Collection

We scrutinised the EMR for all patients, that met the inclusion criteria, for PI‐related diagnoses [23].

**FIGURE 1:**
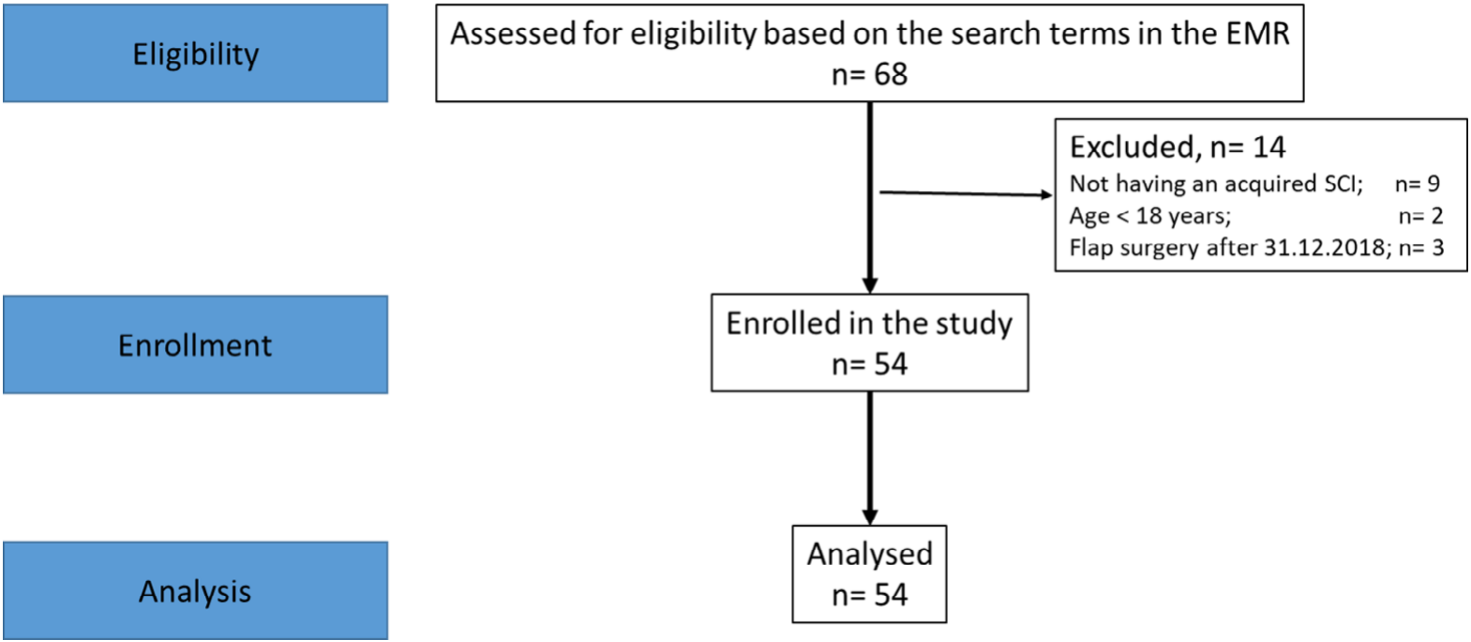
Flowchart of patient inclusion in the study.

The searched data consisted of discharge summaries from the plastic surgeons, admission records from the physician at the SCU, nursing documentation, interdisciplinary wound care records, discharge summaries from the SCU and outpatient summaries. The flowchart of the study is shown in Figure 1.

### 2.4 Study Variables

Study variables were recorded as ‘yes’ if present, ‘no’ if not present and ‘unknown’ if the information was missing from the EMR. Likewise, dichotomous data like gender (male/female) and marital status (living alone/living with a partner) were recorded as zero or one. The term PIR was defined as PI at the same location as the flap surgery was performed. This is in accordance with previous studies [12, 14]. A new PI was categorised in accordance with the classification of the European Pressure Ulcer Advisory Panel [2]. Due to the lack of documentation of category 1 PI in the EMR, only information regarding new PIs in category 2, 3 and 4 was collected [2]. The International Standards for Neurological Classification of SCI were used, including the clinical findings standardised by the American Spinal Injury Association Impairment Scale (AIS) [23, 24]. Relevant information recorded from the EMR was date of birth, date of injury and neurological level of the SCI (cervical, thoracic‐sacral). We furthermore recorded flap techniques, the occurrence of PI and PIR, as well as SCI‐associated problems, such as incontinence, in conjunction with premorbid comorbidities, hypertension, cardiac disease, diabetes mellitus and body mass index (BMI) based on information in the EMR [1, 7, 24, 25]. Information regarding the use of assistive aids (type of wheelchair in use, type of cushion in the wheelchair, mattress at home and transfer aids) were recorded. Support from the wound team at the SCU after discharge was recorded as yes if it involved the patient receiving telemedicine follow‐up at least once a month for 6 months, following flap surgery, and no if not present.

At the end, we recorded support from the district nurses and consumer‐directed assistants (personal assistant follow‐up [PAF]) (yes/no). Due to a lack of information in the EMR, data regarding the time from flap surgery to the onset of PIR is not included in the study. There was also limited information in the EMR regarding the mental and psychological status of the investigated patients, this information was therefore not collected.

### 2.5 Statistical Analyses

Potential risk factors diagnosed before the occurrence of PI were included in the analyses. Continuous variables are presented as mean with standard deviation (SD). Categorical variables are presented as counts and percentages. Categorisation of age into age groups was performed according to the newest SCI recommendations [26, 27]. Participants' demographics and SCI characteristics were analysed descriptively. The term period prevalence refers to PIR in the 10‐year period between 2008 and 2019. Univariate analyses were conducted to examine the distribution within the different categories of each variable. Chi‐square tests (*χ* ^2^) were applied to analyse bivariate associations. Fisher exact probability tests were applied to analyse bivariate variables where it was expected that less than five individuals developed PIR in the group outside the normal range. Logistic regression was used for variables with more than two levels. The analyses were employed to investigate associations between the dependent variable (PIR) and each of the independent variables (potential risk factors). The crude odds ratio (OR) was calculated together with a 95% confidence interval (CI). *p*‐values less than 0.05 were considered significant. The statistical software IBM SPSS Statistics, version 27, was employed for the statistical analyses.

## 3 Results

### 3.1 Demographic Data

We included 54 individuals who underwent flap surgery in the period of interest, 47 of them were men (87%). Most of the individuals had a paraplegia (*n* = 35, 65%), and 45 individuals (83%) had a complete SCI (AIS A). The descriptive baseline data of the population are shown in Table 1.

**TABLE 1:** Baseline data of the population.

Variable	*N* = 54	%
Gender		
Women	7	13
Men	47	87
Age at spinal cord injury	34 years (SD 12.7)	
Years from spinal cord injury to flap surgery	17 years (SD 11.9)	
Neurological level of the spinal injury^a^		
C1–C4	5	9
C5–C8	14	26
T1–S3	35	65
AIS grade^b^		
A	45	83
B	1	2
C	4	7
D	4	7
Marital status		
Living alone	37	69
Married	17	31
Work		
Yes	19	35
No	35	65

Abbreviation: SD, standard deviation.

^a^ The neurological level of the injury in the spinal cord, level C = the cervical part, T = the thoracic part, S = the sacral part.

^b^ AIS = American Spinal Injury Association Impairment Scale, A = sensory and motor complete, B = sensory incomplete and motor complete, C = sensory incomplete and more than half of the key muscle functions below the neurological level of spinal cord injury have a muscle grade less than 3 on the Oxford scale, D = sensory incomplete and more than half of the key muscles below the neurological level of the spinal cord injury have a muscle grade of 3 or greater on the Oxford scale [25].

### 3.2 Pressure Injury Recurrence

**FIGURE 2:**
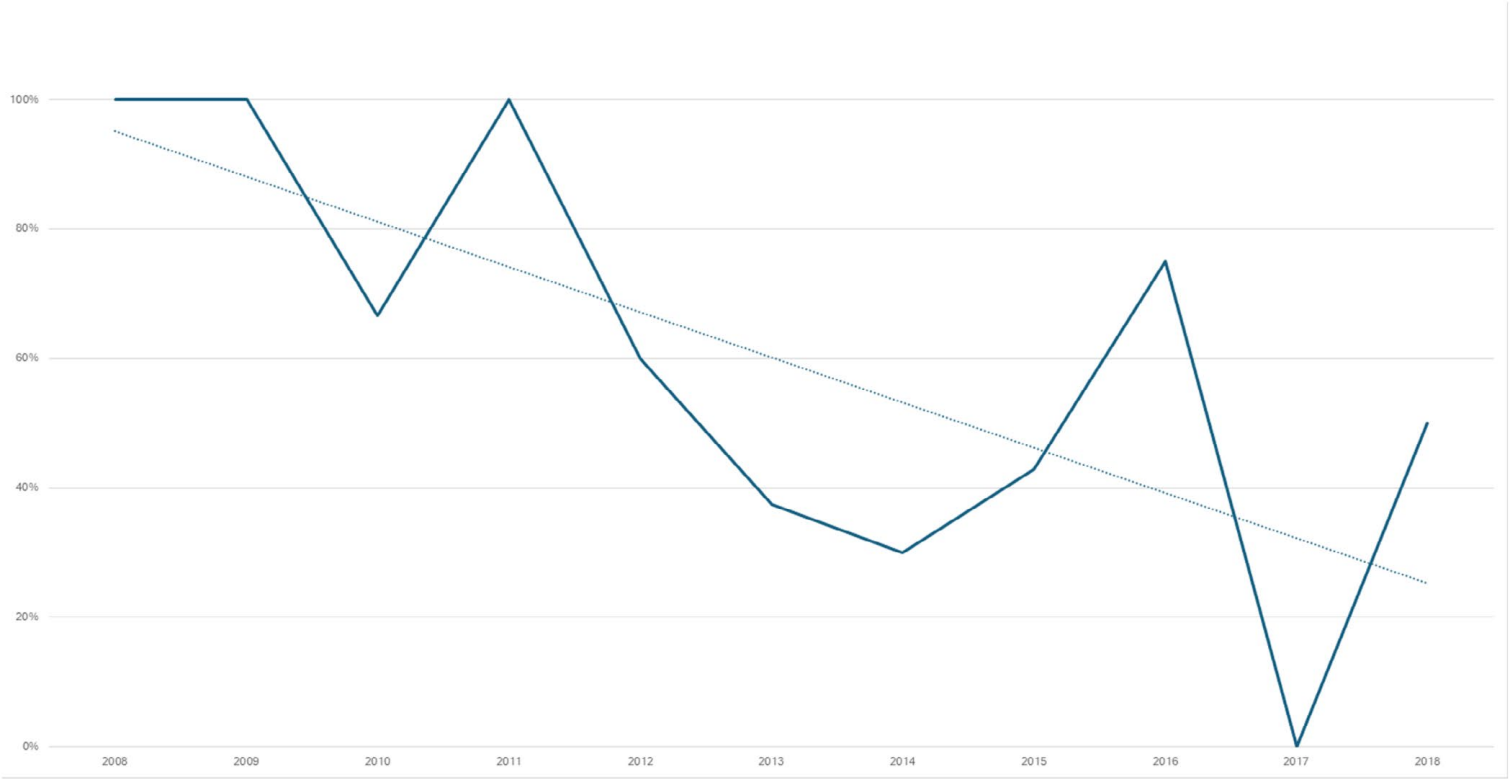
Pressure injury recurrence per year with trendline.

The EMR identified 25 individuals (46%) who developed PIR. However, the period prevalence of PIR decreased significantly over time from 2008 to the end of 2018 (*n* = 54) (OR = 0.7, 95% CI 0.58–0.93, *p* = 0.01), as shown in Figure 2.

The mean age at time of flap surgery was 51 years (SD 12.6). No significant differences were found in the odds of PIR in terms of gender, age when flap surgery was performed, marital status, or whether the patient was working, as shown in Table 2.

**TABLE 2:** Demographics of the investigated population in relation to pressure injury recurrence.

	PIR	OR	95% CI	*p*
Gender				
Women	2 (29%)			
Men	23 (49%)	0.42	0.07–2.41	0.43
Age at spinal cord injury				
0–15	1 (50%)	1.1	0.06–20.21	0.83
16–29	10 (480%)			
30–44	7 (38%)	1.1	0.28–4.26	0.67
45–59	5 (38%)	0.69	0.17–2.81	0.72
60–74	1 (33%)	0.55	0.04–7.04	0.67
Marital status				
Living alone	18 (49%)	1.4	0.42–4.33	0.61
Partner	7 (41%)			
Work				
Yes	12 (63%)	2.9	0.91–9.22	0.07
No	13 (37%)			

Abbreviations: CI, confidence interval; OR, odds ratio; PIR, pressure injury recurrence.

### 3.3 PIR Related to Different Flap Techniques, Previous PI Location and History of Previous PI

We have categorised the 19 different surgical flap techniques described in the EMR, into four groups based on the fundamental method of surgery and the type of tissue used for reconstruction. No significance was found regarding the association between the different groups of flap techniques and PIR as shown in Table 3. A description of the different flap techniques can be found in the Supporting Information.

**TABLE 3:** The technique of flap surgery, history of PI, location of previous PI and the odds of PIR after flap surgery.

	PIR	OR	95% (CI)	*p*
The technique of flap surgery				
Perforator flaps	3 (38%)	0.65	0.14–3.06	0.71
Local flaps	13 (59%)	2.41	0.79–7.31	0.12
Musculocutaneous flaps	3 (50%)	1.18	0.14–9.71	1.00
special techniquesI	1 (17%)	0.2	0.01–2.03	0.12
History of PI				
Yes	21 (57%)	3.94	1.07–14.50	**0.03**
No	4 (25%)			
Localisation of previous PI				
Sacrum	5 (56%)	1.03	0.23–4.58	0.97
Sit bone	15 (68%)	3.67	1.01–13.40	**0.04**
Trochanter major	8 (73%)	3.05	0.67–13.77	0.14
Heal	4 (67%)	1.89	0.23–23.10	0.67
Foot	8 (31%)	2.14	0.53–8.72	0.28
Other locations	4 (50%)	0.83	0.13–5.33	1.00

*Note:* The values in bold show variables with significant associations with PIR. Each of the different flap techniques is compared with all the other flap techniques, perforator flaps, musculocutaneous flaps and specilised techniques were analysed with Fischer exact probability test. Local flaps were analysed with Chi square test. The different body localisations of previous PI are compared with all other body localisations.

Abbreviations: CI, confidence interval; OR, odds ratio; PIR, pressure injury recurrence.

Individuals who had previously experienced PI over the sit bones had higher odds of PIR compared with persons who experienced PI at this body location for the first time (*n* = 15) (OR = 3.4, 95% CI 1.07–14.50, *p* = 0.03). Individuals with PI at the sit bone also had increased odds as compared with individuals who experienced PI in all other assessed body locations (*n* = 39) (OR = 3.7, 95% CI 1.01–13.40, *p* = 0.04). Table 3 shows the different flap techniques, location of previous PI and the odds of PIR after flap surgery.

### 3.4 PIR Related to SCI‐Associated Conditions

Individuals with tetraplegia (C5–C8) had decreased odds of PIR compared with persons with a paraplegic SCI at level T1–S3 (*n* = 3) (OR = 0.18, 95% CI 0.04–0.83, *p* = 0.02). No significant odds were found regarding PIR and the completeness of the SCI (AIS grade). Table 4 shows SCI‐associated conditions and the odds of PIR.

**TABLE 4:** SCI‐associated conditions and PIR.

	PIR	OR	95% CI	*p*
Neurological level of injury				
C1–C4	1 (20%)	0.17	0.02–1.74	0.09
C5–C8	3 (21%)	0.18	0.04–0.83	**0.02**
T1–S3	21 (60%)			
AIS grade				
A, B	22 (48%)	0.52	0.12–2.35	0.48
C, D	9 (60%)			
Spasms/pain				
Yes	21 (45%)	0.81	0.14–4.42	0.81
No	3 (50%)			
Autonomic dysreflexia				
Yes	3 (59%)	0.86	0.11–5.80	0.85
No	20 (31%)			
Incontinence bladder/bowel				
Yes	8 (50%)	0.58	0.38–3.98	0.72
No	17 (45%)			

*Note:* The values in bold show variables with significant associations with PIR. AIS grade was grouped due to a low number of participants, and because the functional levels for individuals with AIS grades A and B are practically similar. AD was analysed with Fischer exact probability test.

Abbreviations: CI, confidence interval; OR, odds ratio; PIR, pressure injury recurrence.

### 3.5 PIR Related to Lifestyle and Comorbidity

Body weight was recorded in the EMR for less than half of the participants. Thirteen (57%) of these persons had a BMI within the recommended range for people with SCI [26]. We found 10 persons (43%) who were either above or under the recommended weight range. No association was found regarding BMI and PIR (*n* = 4) (OR = 0.94, 95% CI 0.13–7.05, *p* = 0.94).

Neither did we find any significant differences for patients with non‐medical comorbidities and the odds of PIR, versus patients with known comorbidities, no matter whether they had one, two, three or four different medical comorbidities.

### 3.6 Assistive Aids After the Flap Surgery

Individuals using a manual wheelchair as their preferred mobility aid post‐surgery had significantly increased odds of PIR, compared with individuals preferring a power wheelchair (*n* = 11) (OR = 3.9, 95% CI 1.06–14.33, *p* = 0.04). Table 5 shows the investigated assistive aids needed by the population post‐flap surgery and the odds of PIR.

**TABLE 5:** Assistive aids and odds of PIR.

	PIR	OR	95% CI	*p*
Powered wheelchair	8 (32%)			
Manual wheelchair	11 (65%)	3.9	1.06–14.33	**0.04**
Air cushion	16 (59%)			
Other cushions	8 (31%)	0.6	0.09–3.83	0.57
Alternating air mattress	17 (47%)			
Other mattresses	6 (60%)	1.04	0.29–0.72	0.95
Transfer board and slide	11 (50%)			
Other/non‐transfer aids	13 (43%)	0.76	0.25–2.31	0.63

*Note:* The values in bold show variables with significant associations with PIR. The values in bold show variables with significant associations with PIR. Other cushions mean gel or foam cushion. Other mattresses mean gel or foam mattress. Other transfer aids mean no transfer aids or patient body lift.

Abbreviations: CI, confidence interval; OR, odds ratio; PIR, pressure injury recurrence.

### 3.7 Local Home Support

Persons needing support to change position had significantly decreased odds of PIR after flap surgery compared with those who were self‐dependent in terms of position change (*n* = 6) (OR = 0.29, 95% CI 0.91–0.95, *p* = 0.04). Individuals receiving support from consumer‐directed assistants (PAF) had significantly decreased odds of PIR compared with those without such support (*n* = 2) (OR = 0.15, 95% CI 0.03–0.76, *p* = 0.01). Furthermore, the odds of PIR were significantly decreased for the group of participants receiving telemedicine follow‐up care from the wound team at the SCU minimum once a month, in at least 6‐month post‐surgery, compared with those with limited or no follow‐up from the SCU (*n* = 5) (OR = 0.28, 95% CI 0.08–0.96, *p* = 0.04). Table 6 shows the findings regarding local home support and the odds of PIR.

**TABLE 6:** Consumer‐directed support and follow‐up from the spinal cord unit (SCU) and the odds of PIR.

	PIR	OR	95% CI	*p*
Assistance in position changes				
Yes	6 (29%)	0.29	0.91–0.95	**0.04**
No	19 (58%)			
Follow‐up from the local homecare service				
Yes	22 (51%)	4.19	0.80–22.06	0.07
No	2 (20%)			
Follow‐up from personal assistants				
Yes	2 (15%)	0.15	0.03–0.76	**0.01**
No	22 (55%)			
Follow‐up from the SCU via telemedicine^a^				
Yes	5 (26%)	0.28	0.08–0.96	**0.04**
No	19 (56%)			

*Note:* The values in bold show variables with significant associations with PIR.

Abbreviations: CI, confidence interval; OR, odds ratio; PIR, pressure injury recurrence.

^a^ Continuous follow‐up via the interdisciplinary wound team at the spinal cord unit via videoconference consultations and in cooperation with the district nurses in the local homecare service.

## 4 Discussion

This is the first Norwegian study in the population of individuals with SCI that investigates the period prevalence of PIR and potential risk factors for PIR‐development, post‐surgery. Based on the annual number of flap surgeries among people with SCI in Norway, the number of participants included appears to be representative of the population.

In the current study, we identified 54 individuals treated with flap surgery between 2008 and 2019. Nearly half of the participants experienced a PIR post‐surgery. Although the period prevalence is high, the prevalence of PIR is similar to the findings in international studies [12, 14, 15]. However, in the current study, the PIR trendline shows a decline from 2008 to 2019. A potential explanation may be that individuals who underwent surgery in 2008 had more time to develop PIR compared with persons who had their surgery in 2018. The reduction in PIR may also be related to the establishment of the national patient safety programme [28]. Furthermore, the establishment of a multidisciplinary outpatient wound clinic at the SCU, increased the focus on prevention and treatment of PI and PIR after flap surgery. In addition, the results might be due to improved surgical techniques and better methods to identify patients suitable for PI surgery [17, 19]. However, no differences were found between different flap techniques and PIR in this study. Bates‐Jensen et al. (2009) found that most PIR occurred within 4 months after flap surgery. Bates‐Jensen et al. (2009) found that most PIR occurred within 4 months after flap surgery [8]. However, further research on the time from flap surgery to the onset of PIR is warranted.

This study showed that individuals using a manual wheelchair had increased odds of PIR compared with persons using a power wheelchair. Decreased odds of PIR were observed in persons with tetraplegia compared with those with paraplegia. Furthermore, decreased odds were found in individuals needing support in position changing compared with those able to change position independently. These findings are somewhat surprising, considering that lack of independence in changing positions is considered as one of the most significant causes of PI development [2, 27]. However, this might be related to the finding regarding receiving support from a PAF was associated with a decreased risk of PIR. PAF is a public service in the municipalities, which provides practical and individualised support to people with severe disabilities, aiming to enhance their participation and activity in daily life situations [29, 30]. The person in need of support will be the employer of the PAF and will have a few employees who ensure customisation of routines for position change, skin inspection and use of preventive aids, according to the person's specific needs. This increases the opportunities for continuity and customised care, which are both crucial for reducing the risk of PI, as well as reducing the risk of other SCI‐associated conditions [29, 30]. We therefore speculate whether continuous follow‐up and care by a small number of persons who are trained in PIR prevention may explain why people in greater need of support and assistance have decreased odds of PIR, compared with individuals without such support? Further research on this topic is warranted.

Video conference follow‐up by the wound clinic at the SCU in collaboration with the district nurses was associated with reduced odds of PIR. We speculate that follow‐up by the SCU via video conference may be another way to ensure continuity in care and person‐centred follow‐up after flap surgery, since it involves follow‐up by the same wound care nurse over time. Furthermore, video conference follow‐up may also increase collaboration and knowledge exchange regarding PI and SCI between the SCU, the patients and the district nurses or PAFs [31, 32]. Previous research has shown telemedicine to be as effective as traditional follow‐up in terms of PI healing, health‐related quality of life, user satisfaction and cost [31, 32]. The increased use of telemedicine in patient care and treatment is expected to continue [20]. However, it is essential to ensure a person‐centred approach, and that the technology is used to benefit the patient, and not the contrary [20, 32]. A person‐centred approach lays the foundation for effective collaboration between patients, their families and healthcare professionals, based on mutual respect [33]. This approach allows for the development of personalised solutions that best meet the needs of each patient [33]. Thus, more research focusing on the impact of telemedicine in the prevention of PIR after flap surgery is warranted.

Follow‐up solely by the local homecare service did not yield the same good results as follow‐up by PAFs and the multidisciplinary wound team at the SCU. This could be due to a high turnover and many part‐time employees among the district nurses, as well as lack of sufficient documentation in the EMR, which might lead to a lack of continuity in patient care [34, 35]. Inconsistency in the care might impede the detection of changes in the operated area during skin inspections or impede identification of any need for changes in the ongoing preventive measures.

Sufficient documentation is also crucial for the transition of the patient from the hospital to home, to ensure a comprehensive PIR prevention after discharge from the postoperative stay at the SCU [28, 35]. We found that there was a lack of documentation related to post‐flap surgery follow‐up at the SCU. Less than half of the patients had their nutrition status or BMI recorded during the postoperative stage. In addition, there was insufficient documentation regarding the use of stimulants and of any mental health challenges, so that we could not include variables related to this topic in the study.

A lack of proper and accurate documentation in the EMR may contribute to a less streamlined transition between hospital and home, [31, 35] hinder person‐centred care and compromise patient safety, as a worst‐case scenario [28, 35].

Individuals with a history of previous PI, especially PI over the sit bone area, had increased odds of having a new PIR, compared with those experiencing PI for the first time.

Possible causes of PIR may include failure to identify the cause of pressure ulcer development and a lack of knowledge or routines for inspecting and managing vulnerable skin areas, either independently or with healthcare assistance [1, 3, 5]. Due to impaired skin sensation and the inability to detect PIR, regular visual skin inspections are necessary. Training in the use of cameras or mirrors to inspect the operated body area is crucial, as it enhances the patient's ability for self‐care as well as the possibility to detect PI early and thus, enable prompt preventive measures to avoid severe recurrence [1, 3, 5]. Effective routines and knowledge about how to perform safe transfers is crucial to avoid friction and shear forces at the operated body location [1, 3, 5]. Moreover, the patients and healthcare professionals' knowledge about PI prevention and the motivation to follow recommended measures are significant [14]. Garber et al. points out that some individuals with a history of PI do not believe that they are at risk for developing new PI's [1, 3, 5]. A person‐centred approach may potentially help motivate the patients and implement preventive measures tailored to the patient's specific needs, effectively reducing the likelihood of PIR by addressing specific risk factors which may cause recurrent PI at the same body location [31].

Based on findings from this study and related research on the association between PIR and previous pressure injuries at the sit bones [12, 13, 14], we speculate that pressure injuries at the sit bones might serve as a predictor for PIR. However, further research is warranted.

A person‐centred approach may also be essential in the selection and use of assistive aids. Most of the patients in the current study used appropriate cushions and mattresses, as recommended by the interdisciplinary team at the SCU, and in line with evidence‐based guidelines regarding PI prevention [24, 36]. But the findings showed increased odds of PIR in individuals using a manual wheelchair, as compared with those using a power wheelchair post‐surgery. This might be due to the possibility of relieving the body load on the operated area when using a power wheelchair, because of the tilt function in the power wheelchair, as well as the possibility to ensure a safe transfer between the bed and wheelchair [1, 36, 37]. However, this topic should be investigated more thoroughly.

Despite the benefits mentioned, many people with SCI prefer to use a manual wheelchair because they value the physical activity they can perform via the chair, and the choice of wheelchair may be connected to their identification as abled or disabled [1, 3, 5]. Furthermore, increased physical activity due to using a manual wheelchair increases the body blood flow and thereby tissue oxygenation, potentially reducing the odds of PIR [36]. In addition, individuals using manual wheelchairs generally report greater satisfaction versus people using power wheelchairs [36]. This further highlights the importance of considering individual preferences and a person‐centred approach in PIR prevention.

No association was identified between bladder and bowel incontinence and PIR. This is not in accordance with the findings from a previous study, where an association was found between incontinence and PI development [7, 38]. The results of the current study may be influenced by the unique context of the Norwegian healthcare system, where governmental funding ensures access to adequate incontinence aids [39]. However, a previous Norwegian study showed that incontinence was associated with increased risk of PI in people with a newly acquired SCI [7]. This suggests a continued need for enhanced information and support for home‐dwelling patients and their local healthcare providers regarding available treatment options and skincare.

### 4.1 Limitations

The non‐randomised nature of the study limits the ability to estimate causal effects.

Even though the number of participants seems to be representative of the population of people with SCI in Norway, the total number is still small. This may result in difficulties finding significant differences in this population. It was also a limitation that we were not able to perform multivariate analysis due to insufficient data and the small sample size.

Category 1 PI [24] is known to be insufficiently mentioned in the EMR, and category 1 PIR was accordingly not found in the investigated data. Lack of documentation regarding variables such as tobacco use and BMI, as well as lack of collected data on time from flap surgery to PIR, and median follow‐up time, are other limitations. This could provide important insight regarding the characteristics for PIR and should be investigated further.

Due to the lack of information in the EMR, the current study did not thoroughly explore psychosocial factors and compliance, including the patients' motivation and mental health. A previous study found these variables to affect the potential risk of developing PI [7]. Therefore, this information needs to be considered in any future development of a holistic PIR prevention programme.

### 4.2 Implications for Practice

The study highlights the need for further research to develop a more comprehensive understanding of the factors influencing PIR after flap surgery.

Prevention of PIR after flap surgery in home‐dwelling patients with SCI is crucial. Ensuring continuity in the follow‐up care and support with position changes and skin inspections, particularly by trained personnel, may potentially reduce the odds of PIR.

The establishment of interdisciplinary wound clinics at the SCUs, together with increased collaboration between the SCUs and the community healthcare services, could improve continuity of care and knowledge exchange. The use of telemedicine in this cooperation will be beneficial and can enhance PIR prevention. However, a person‐centred care is essential to ensure that the follow‐up is tailored to fit each person.

Adequate documentation is crucial to ensure continuity of care in the homecare service and at the SCU, to ensure safe transfers between hospital and home. The provision of a comprehensive, knowledge‐based education program to patients, their relatives and the local healthcare providers regarding PIR prevention can support informed decision‐making, and thus ensure person‐centred care and adherence to preventive measures. The development of a PIR prevention programme that addresses identified risk factors can help to reduce the occurrence of PIR. This includes ensuring that patients have access to appropriate assistive devices and person‐centred follow‐up.

## 5 Conclusion

The current retrospective, single‐centre study reveals a high period prevalence of PIR after flap surgery in the home‐dwelling population of people with SCI in Norway. Tetraplegia, need for support in position change, use of power wheelchair, follow up from PAFs and the SCU post‐surgery, were associated with reduced odds of PIR. The findings should be applied to the development of a PIR prevention programme, although increased understanding of PIR risk factors requires additional studies.

## Ethics Statement

The Norwegian Data and Telecommunications Authority's safe information flow requirements were followed and the study was approved by the Norwegian Agency for Shared Services in Education and Research (SIKT/2020/113986). There was no need for ethical approval by the National Regional Ethical Committee, as this study was an internal quality improvement project. The study is performed in accordance with the Helsinki Declaration.

## Consent

Since this is an internal quality improvement project, the study has been granted an exemption requirement for patient consent (SIKT/2020/113986).

## Conflicts of Interest

The authors declare no conflicts of interest.

## Supporting information


**Data S1** Supporting Information.

## Data Availability

Data available on request, in accordace with the national GDPR legislation.
