# Supplementary material for: Pressure Injury Recurrence After Flap Surgery in Home‐Dwelling Patients With Spinal Cord Injury in Norway: A Retrospective Study
Source: Int Wound J. 2025 Apr 15;22(4):e70211. doi: 10.1111/iwj.70211 (PMC11999728; doi:10.1111/iwj.70211)
Supplement: Supplementary file 1 — Data S1 Supporting Information. [file IWJ-22-e70211-s001.doc]

**Flap techniques**

The 19 different surgical flap-techniques described in the EPR, can be categorized into four groups based on the fundamental method of surgery and the type of tissue used for reconstruction.

**1. Perforator Flaps** (n= 8) focus on minimizing donor site morbidity while providing flexibility for reconstruction. These flaps use skin and subcutaneous tissue supplied by perforating blood vessels (without including muscle). Three specific techniques were found

Perforator flap (n= 6)

Pedicled DIEP flap (n=1)

Pedicled eye-gap perforator (n= 1)

**2. Local Flaps** (n= 22) are straightforward techniques suitable for small to medium-sized defects. These are flaps mobilized from adjacent areas without significant displacement, relying on local blood supply. Five specific techniques were found

VY technique (n= 16)

Local flap (n= 2)

Rotation flap (n= 2)

Transposition flap (S GAP) (n= 2)

**3. Musculocutaneous Flaps** (n= 12) offer better coverage and blood supply for more complex cases. These flaps combine muscle and skin as a single unit to cover complex wounds. Three specific techniques were found.

Musculocutaneous flap (n= 4)

Tensor fascia lata (n= 3)

Pedicled latissimus dorsi flap (n=5)

**4. Specialized Techniques and Filet Flaps** (n= ) involve technical variations designed for specific needs. This category includes flaps with more specific techniques or anatomical adaptations. Three specific techniques were found.

Filet flap (n= 1)

Adipocutaneous flap (n= 1)

Fasciocutaneous flap in GAP (n= 3)

**5. Missing data** (n= 11)
